# *TERT* rs10069690 polymorphism and cancers risk: A meta‐analysis

**DOI:** 10.1002/mgg3.903

**Published:** 2019-08-27

**Authors:** Guisheng He, Tao Song, Yazhen Zhang, Xiuxiu Chen, Wei Xiong, Huamin Chen, Chuanwei Sun, Chaoyang Zhao, Yunjing Chen, Huangfu Wu

**Affiliations:** ^1^ Department of Surgical Oncology Second Affiliated Hospital of Hainan Medical College Haikou Hainan Province China

**Keywords:** cancer, meta‐analysis, polymorphism, *TERT*

## Abstract

**Background:**

Studies have identified that the telomerase reverse transcriptase (*TERT*) gene polymorphism rs10069690 (C>T) is associated with cancer risk, but the results remain inconclusive.

**Methods:**

To provide a more precise estimation of the relationship, we performed a meta‐analysis of 45 published studies including 329,035 cases and 730,940 controls. We conducted a search in PubMed, Google Scholar and Web of Science to select studies on the association between rs10069690 and cancer risk. Stratification by ethnicity, cancer type, cancers’ classification, source of control, sample size, and genotype method was used to explore the source of heterogeneity. The pooled odds ratios (ORs) and corresponding 95% confidence intervals (CIs) were evaluated using random effects models. Sensitivity, publication bias, false‐positive report probability (FPRP) and statistical power were also assessed.

**Results:**

The result demonstrated that rs10069690 was significantly associated with an increased risk of cancer overall (OR = 1.09, 95% CI: 1.06–1.12, *p* < .001) under the allele model. Stratification analysis revealed an increased cancer risk in subgroups of breast cancer, ovarian cancer, lung cancer, thyroid cancer, and renal cell carcinoma (RCC). However, a significantly decreased association was observed in pancreatic cancer in the European population (OR = 0.93,95% CI: 0.87–0.99, *p* = .031). In the subgroup analysis based on cancer type, no significant association was found in prostate cancer, leukemia, colorectal cancer and glioma.

**Conclusions:**

This meta‐analysis suggested that the *TERT* rs10069690 polymorphism may be a risk factor for cancer, especially breast cancer, ovarian cancer, lung cancer, thyroid cancer, and RCC. Further functional studies are warranted to reveal the role of the polymorphism in carcinogenesis.

## 1 INTRODUCTION

Cancer is one of the leading causes of human death worldwide and constitutes an enormous burden on the society in both economically developed and developing countries (Are et al., 2013). Based on GLOBOCAN estimates, about 18.1 million newly diagnosed cancer patients and 9.6 cancer million deaths occurred in 2018 worldwide (Bray et al., 2018). The mechanism of occurrence and development of cancer remains unclear. People generally agree that a complex interaction between genetic and environmental factors may contribute to cancer development. Recently, genome‐wide association studies (GWAS) have demonstrated that single nucleotide polymorphisms (SNPs) in Chromosome 5p15.33, which is a crucial genomic region for telomere biology and contains two well‐known genes: telomerase reverse transcriptase (TERT) and cleft lip and palate transmembrane 1‐like (CLPTM1L), are significantly associated with cancer risk (Bojesen et al., 2013; Haiman et al., 2011; Rafnar et al., 2009; Wolpin et al., 2014).

Telomeres consisting of TTAGGG repeats are specialized structures at the end of eukaryotic chromosomes that protect chromosomes from degradation, end‐to‐end fusion, and atypical recombination; thus, telomeres are crucial in maintaining chromosome integrity and genomic stability (Blackburn, 2005). Telomere length is maintained by telomerase, a ribonucleoprotein enzyme that adds the telomeric repeat sequence directly to the single‐strand 3’ overhang to maintain telomere ends that have been incrementally shortened by each cell division (Collins & Mitchell, 2002). The expression of telomerase is extremely low in most normal human somatic cells, but is present in over 90% of human malignancies. As the catalytic subunit of telomerase, TERT is the most important determinant in the regulation of telomerase expression (Zhang et al., 2000).

*TERT*, located on the short (p) arm of chromosome 5 at position 15.33 (5p15.33), encodes a catalytic subunit of telomerase and exerts a pivotal role in the maintenance of telomere DNA length and carcinogenesis. Mutations in the coding regions of *TERT* can affect telomerase activity and telomere length, and generate severe clinical phenotypes, including a substantive increase in cancer frequency (Baird, 2010). Previous studies have demonstrated that rs10069690 (C>T) polymorphism in the *TERT* is associated with susceptibility to multiple types of cancer, such as breast cancer (Bojesen et al., 2013; Haiman et al., 2011; Huo et al., 2016; Michailidou et al., 2015, 2017), ovarian cancer (Bojesen et al., 2013; Earp et al., 2016; Kuchenbaecker et al., 2015; Lee et al., 2016; Phelan et al., 2017), lung cancer (Landi et al., 2009; Ye et al., 2017), and thyroid cancer (Gong et al., 2016; Gudmundsson et al., 2017). However, studies have yet to reach a consensus.

Meanwhile, a single study might have been underpowered to detect the overall effects. A quantitative synthesis of the accumulated data from different studies is important to provide evidence on the association of rs10069690 polymorphism with cancer risk. Therefore, in this study, we performed a comprehensive meta‐analysis including the latest and relevant articles to explore the association between the *TERT* rs10069690 polymorphism and cancer risk.

## 2 METHODS

### 2.1 Search strategy

According to the Meta‐analysis of Observational Studies in Epidemiology guidelines, we performed a systematic literature search on PubMed, Google Scholar, Embase, Web of Science, China national knowledge infrastructure (CNKI) and Wan fang electronic databases and sample size limitations covering all publications regarding the association between TERT polymorphisms and cancer susceptibility up to the end of May 2019. The search terms were as follows: “TERT”, “telomerase reverse transcriptase”, “5p15”, “polymorphism”, ‘“SNP”’, “variant’’, “cancer”, “tumor” “carcinoma” and ‘“malignancy”’. The search was limited to English language papers and human studies. In addition, references of articles and reviews were also searched to find other eligible studies. When an article reported results on different subpopulations, we treated each subpopulation as a separate comparison.

### 2.2 Inclusion and exclusion criteria

In this meta‐analysis, the following inclusion criteria were used for selecting the studies: (a) population‐ or hospital‐based case–control studies published in English as original articles; (b) investigating *TERT* rs10069690 polymorphism and cancer susceptibility; (c) studies provided the odds ratios (OR) estimates and their 95% confidence intervals (CIs) in allele model. The exclusion criteria were: (a) not involving TERT and rs10069690 polymorphism research; (b) case reports, reviews, repeated literature, nonhuman studies; (c) no available data presented.

### 2.3 Data extraction

Two investigators independently extracted the data from all eligible publications, according to the inclusion and exclusion criteria listed above. Discrepancies were resolved by discussion and consensus. We extracted the following information from each study when available: the first author's last name, year of publication, cancer type, patient ethnicity, number of cases and controls, genotyping method, the odds ratios (ORs) estimates and their 95% confidence intervals (CIs) in allele model. Quality scores of studies ranged from 0 (lowest) to 15 (highest). Studies with scores ≤9 were categorized into low quality, while those with scores >9 were considered as high quality (Fu et al., 2017).

### 2.4 Statistical analysis

We used the ORs with 95% CIs to assess the strength of association between the *TERT* rs10069690 polymorphism and cancers risk. The OR and the 95% CI in each comparison were assessed in the allele model. Stratified analyses were performed by cancer type (if one cancer type contained less than two individual studies, it was combined into the “other cancers” group), ethnicity, sample size, and genotyping method under the allele model. Heterogeneity was checked using the Chi‐square‐based Q statistic test. If the result of heterogeneity test was *p* > .05, then the pooled ORs were calculated using the fixed‐effects model with the Mantel–Haenszel method. If heterogeneity was present (*p* < .05), the random effects model (the DerSimonian and Laird method) was selected. The literature publication bias was estimated using the Funnel plot and Egger's linear regression test (*p* < .05 was considered a significant publication bias). The false‐positive report probability (FPRP) was calculated to evaluate the significant findings. We set 0.2 as an FPRP threshold and assigned a prior probability of 0.1 to detect an odds ratio (OR) of 0.67/1.50 (protective/risk effects) for an association with cancer risk under investigation. Only the significant result with an FPRP value less than 0.2 was considered a noteworthy finding (He et al., 2013). All statistical analyses were conducted using the Stata software (version 11.0; Stata Corporation), using two‐sided *p* values.

## 3 RESULTS

### 3.1 Characteristics of studies

**Figure 1:**
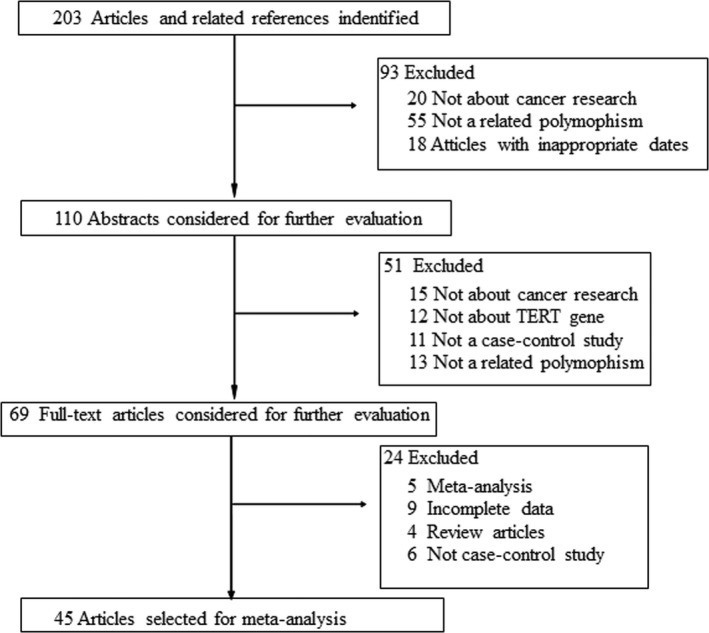
Study inclusion and exclusion procedures

The detailed process of study selection is summarized in the flow diagram (Figure 1). According to the inclusion criteria, a total of 45 eligible studies involving 329,035 cases and 730,940 controls were included in this meta‐analysis. The characteristics of selected studies are summarized in Table1. The 45 studies included nine on breast cancer (Garcia‐Closas et al., 2013; Haiman et al., 2011; Huo et al., 2016; Michailidou et al., 2015, 2017; Palmer et al., 2013; Purrington et al., 2014) and, six on ovarian cancer (Bojesen et al., 2013; Earp et al., 2016; Kuchenbaecker et al., 2015; Lee et al., 2016; Phelan et al., 2017; Terry et al., 2012); five on lung cancer (Gao et al., 2014; Landi et al., 2009; Wang et al., 2016; Ye et al., 2017; Zhao et al., 2013). two each on glioma (Melin et al., 2017; Zhao et al., 2012), thyroid cancer (Gong et al., 2016; Gudmundsson et al., 2017), pancreatic cancer (Campa, Rizzato, et al., 2015; Petersen et al., 2010), prostate cancer (Panagiotou et al., 2015; Schumacher et al., 2011), colorectal cancer (Li et al., 2017; Pellatt, Wolff, Herrick, Lundgreen, & Slattery, 2013), RCC (Martino et al., 2016; Wu, Yan, et al., 2017), and leukemia (Sheng et al., 2013; Speedy et al., 2014); and one each on endometrial cancer (Prescott, McGrath, Lee, Buring, & De Vivo, 2010), bladder cancer (Rothman et al., 2010), testicular germ cell tumor (TGCTs) (Schumacher et al., 2013), melanoma (Llorca‐Cardenosa et al., 2014), multiple myeloma (Campa, Martino, et al., 2015), gastrointestinal stromal tumors (GISTs) (Zhang et al., 2015), non‐Hodgkin's lymphoma (NHL), diffuse Large B‐cell lymphoma (DLBCL), small lymphocytic lymphoma/chronic lymphocytic leukemia (SLL/CLL) (Shadrina et al., 2015), nasopharyngeal carcinoma (NPC) (Zhang et al., 2016), gastric cancer (Duan et al., 2016), esophageal cancer (Wu, Yan, et al., 2017), gastric cardia adenocarcinoma (GCA) (Zhang et al., 2019), hepatocellular carcinoma (HCC) (Zhang et al., 2017). One study focused on Caucasians (Prescott et al., 2010); two studies focused on Africans (Huo et al., 2016; Long et al., 2013); three studies on African‐Americans (Bojesen et al., 2013; Haiman et al., 2011; Palmer et al., 2013), 16 studies on Asians (Bojesen et al., 2013; Duan et al., 2016; Gao et al., 2014; Gong et al., 2016; Li et al., 2017; Sheng et al., 2013; Wang et al., 2016; Wu, Yan, et al., 2017; Wu, Zhu, et al., 2017; Zhang et al., 2017; Ye et al., 2017; Zhang et al., 2019; Zhang et al., 2015; Zhang et al., 2016; Zhao et al., 2012; Zhao et al., 2013); twenty studies on European (Campa, Martino, et al., 2015; Campa, Rizzato, et al., 2015; Earp et al., 2016; Garcia‐Closas et al., 2013; Gudmundsson et al., 2017; Haiman et al., 2011; Kuchenbaecker et al., 2015; Landi et al., 2011; Llorca‐Cardenosa et al., 2014; Martino et al., 2016; Melin et al., 2017; Michailidou et al., 2015, 2017; Mosrati et al., 2015; Panagiotou et al., 2015; Petersen et al., 2010; Prescott et al., 2010; Rothman et al., 2010; Schumacher et al., 2013; Shadrina et al., 2015), and six studies on multiple populations (Lee et al., 2016; Pellatt et al., 2013; Purrington et al., 2014; Schumacher et al., 2011; Speedy et al., 2014; Terry et al., 2012). The studies used genotyping methods such as Illumina, TaqMan, MassArray, Agarose gel electrophoresis, KASP technology, and polymerase chain reaction‐restriction fragment length polymorphism (PCR‐RFLP).

**Table 1:** Study characteristics of the association between the rs10069690 polymorphism and cancer risk in this meta‐analysis

Study (y)	Cancer type	Ethnicity	Method	Source of control	Case	Control	OR (95% CI)	Score
Zhang et al. (2019)	GCA	Asian	MassArray	HB	1,024	1,118	1.42 (1.22–1.66)	10
Gudmundsson et al. (2017)	Thyroid cancer	European	Illumina	Multiple	3,001	287,550	1.20 (1.12–1.29)	12
Michailidou et al. (2017)	Breast cancer	European	Illumina	Multiple	61,282	45,494	1.06 (1.04–1.08)	13
Zhang et al. (2017)	HCC	Asian	MassArray	HB	473	564	0.75 (0.59–0.96)	8
Wu, Yan, et al. (2017)	Esophageal cancer	Asian	MassArray	HB	386	495	1.70 (1.33–2.18)	6
Ye et al. (2017)	Lung cancer	Asian	MassArray	PB	554	603	1.41 (1.14–1.76)	8
Wu, Zhu, et al. (2017)	RCC	Asian	MassArray	PB	293	459	1.39 (1.07–1.81)	6
Melin et al. (2017)	Glioma	European	Illumina	Multiple	1591	804	1.40 (1.20–1.63)	10
Phelan et al. (2017)	Ovarian cancer	European	Illumina	Multiple	16,924	68,502	1.08 (1.05–1.11)	14
Kuchenbaecker et al. (2015)	Ovarian cancer	European	Illumina	Multiple	30,845	9,627	1.14 (1.10–1.19)	12
Li et al. (2017)	Colorectal cancer	Asian	MassArray	PB	247	300	1.30 (0.94–1.80)	5
Lee et al. (2016)	Ovarian cancer	Multiple	Illumina	PB	1,414	4,051	1.14 (1.03–1.26)	13
Earp et al. (2016)	Ovarian cancer	European	Affymetrix	Multiple	3,573	5,640	1.14 (1.06–1.22)	12
Martino et al. (2016)	RCC	European	Agarose gel electrophoresis	PB	243	420	1.20 (0.93–1.55)	5
Zhang et al. (2016)	NPC	Asian	MassArray	PB	855	1,036	1.16 (0.96–1.41)	10
Wang et al. (2016)	Lung cancer	Asian	MassArray	HB	228	301	1.34 (0.98–1.83)	6
Duan et al. (2016)	Gastric cancer	Asian	MassArray	HB	302	300	1.56 (1.15–2.11)	6
Huo et al. (2016)	Breast cancer	African	Illumina	PB	6,657	7,713	1.13 (1.07–1.19)	13
Gong et al. (2016)	Thyroid cancer	Asian	PCR‐RFLP	HB	452	452	1.38 (1.10–1.72)	7
Zhang et al. (2015)	GISTs	Asian	TaqMan	HB	300	300	1.40 (1.04–1.88)	6
Michailidou et al. (2015)	Breast cancer	European	TaqMan	Multiple	62,533	60,976	1.06 (1.04–1.09)	14
Campa, Rizzato, et al. (2015)	Multiple myeloma	European	TaqMan	PB	2,267	2,796	0.88 (0.79–0.97)	11
Shadrina et al. (2015)	NHL	European	TaqMan	PB	344	893	1.01 (0.83–1.24)	8
Shadrina et al. (2015)	DLBCL	European	TaqMan	PB	139	893	0.85 (0.63–1.16)	7
Shadrina et al. (2015)	SLL/CLL	European	TaqMan	PB	77	893	1.21 (0.84–1.73)	5
Campa, Rizzato, et al. (2015)	Pancreatic cancer	European	Illumina	Multiple	1901	4,106	0.95 (0.87–1.05)	12
Panagiotou et al. (2015)	Prostate cancer	European	Illumina	Multiple	23,631	24,534	1.15 (1.12–1.19)	13
Speedy et al. (2014)	Leukemia	Multiple	Illumina	Multiple	2,883	8,350	1.03 (0.96–1.10)	11
Llorca‐Cardenosa et al. (2014)	Melanoma	European	KASPtechnology	HB	648	381	1.02 (0.83–1.23)	8
Gao et al. (2014)	Lung cancer	Asian	MassArray	HB	309	310	1.28 (0.96–1.71)	6
Long et al. (2013)	Breast cancer	African	Illumina	Multiple	1,112	930	0.86 (0.75–0.97)	11
Palmer et al. (2013)	Breast cancer	African‐American	MassArray	Multiple	1,199	1948	1.05 (0.94–1.17)	12
Garcia‐Closas et al. (2013)	Breast cancer	European	Illumina	Multiple	4,193	35,194	1.15 (1.11–1.20)	14
Purrington et al. (2013)	Breast cancer	Multiple	Illumina	Multiple	3,677	4,708	1.24 (1.14–1.34)	11
Bojesen et al. (2013)	Breast cancer	European	Illumina	Multiple	46,451	42,599	1.06 (1.04–1.08)	14
Bojesen et al. (2013)	Breast cancer	Asian	Illumina	Multiple	6,269	6,624	1.04 (0.98–1.10)	13
Bojesen et al. (2013)	Breast cancer	African‐American	Illumina	Multiple	1,116	932	1.19 (1.05–1.35)	10
Bojesen et al. (2013)	Ovarian cancer	European	Illumina	Multiple	986	23,491	1.33 (1.20–1.47)	12
Bojesen et al. (2013)	Ovarian cancer	European	Illumina	Multiple	8,371	23,491	1.15 (1.11–1.20)	13
Zhao et al. (2013)	Lung cancer	Asian	SNPscanTM	PB	784	782	1.14 (0.98–1.32)	9
Pellatt et al. (2013)	Colorectal cancer	Multiple	Illumina	PB	2,309	2,915	1.06 (0.97–1.15)	12
Schumacher et al. (2013)	TGCTs	European	TaqMan	Multiple	940	1559	0.66 (0.53–0.82)	10
Terry et al. (2012)	Ovarian cancer	Multiple	TaqMan	Multiple	2,112	2,456	1.11 (1.00–1.23)	11
Sheng et al. (2013)	leukemia	Asian	TaqMan	HB	570	673	1.27 (1.04–1.56)	10
Haiman et al. (2011)	Breast cancer	African‐American	Illumina	Multiple	1,002	2,743	0.76 (0.68–0.84)	12
Haiman et al. (2011)	Breast cancer	European	Illumina	Multiple	5,007	17,965	1.19 (1.13–1.25)	14
Zhao et al. (2011)	Glioma	Asian	MassArray	HB	983	1,024	0.99 (0.84–1.18)	12
Schumacher et al. (2011)	Prostate cancer	Multiple	Illumina	Multiple	2,782	4,458	0.80 (0.73–0.89)	11
Petersen et al. (2010)	Pancreatic cancer	European	Illumina	Multiple	3,851	3,934	0.91 (0.83–1.00)	10
Rothman et al. (2010)	Bladder cancer	European	Illumina	Multiple	3,532	5,120	0.90 (0.84–0.96)	12
Prescott et al. (2010)	Endometrial cancer	Caucasian	TaqMan	PB	674	1685	1.08 (0.92–1.26)	10
Landi et al. (2009)	Lung cancer	European	Illumina	Multiple	5,739	5,848	1.02 (0.95–1.10)	13

Abbreviations: 95% CI: 95% confidence interval; DLBCL, diffuse Large B‐cell lymphoma; GCA, gastric cardia adenocarcinoma; GISTs, gastrointestinal stromal tumors; HB, hospital based; HCC, hepatocellular carcinoma; NHL, non‐Hodgkin's lymphomas; NPC, nasopharyngeal carcinoma; OR, odds ratio; PB, population based; PCR‐RFLP, polymerase chain reaction‐restriction fragment length polymorphism; RCC, renal cell carcinoma; SLL/CLL, small lymphocytic lymphoma/chronic lymphocytic leukemia; TGCTs, testicular germ cell tumors.

### 3.2 Association between rs10069690 polymorphism and cancer risk

**Figure 2:**
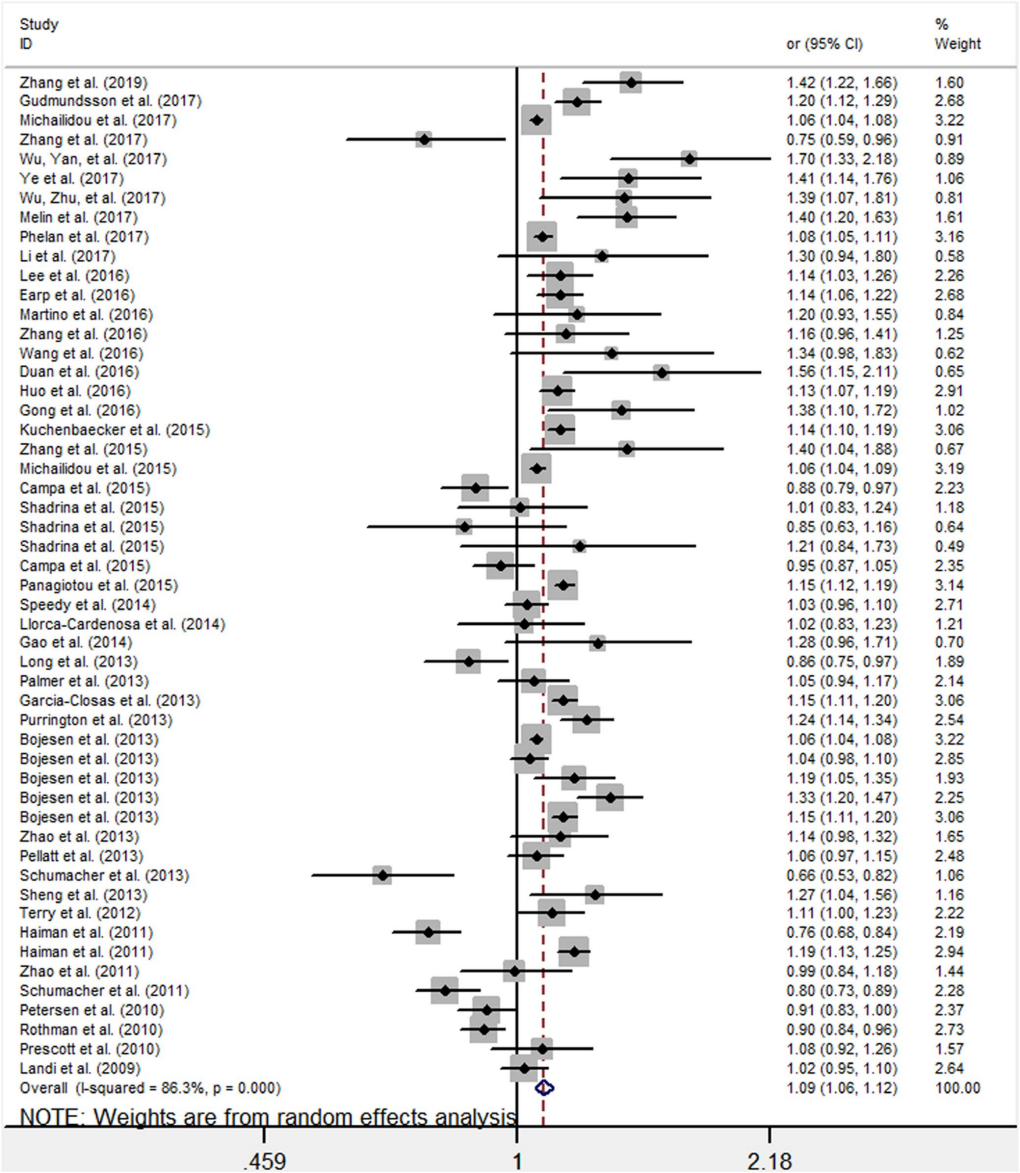
Forest plot of the ORs for the overall cancer risk associated with the *TERT* variant rs10069690 polymorphism

Based on the data from all 45 studies, we found a significant increased cancer risk for the *TERT* rs10069690 under a per‐allele risk analysis (OR = 1.09, 95% CI: 1.06–1.12, *p* < .001), with a statistical power of 100%. The results from a random effect model showed significant heterogeneity (*p*‐heterogeneity < .001, *I* ^2^ = 86.3%) (Figure 2 and Table 2).

**Table 2:** Stratified analyses of the rs10069690 polymorphism and cancer risk

Category	No.	Cases/Controls	OR (95% CI)	*P*	*I* ^2^ (%)	*P*‐ heterogeneity	*P*‐ egger	Power (%)	Prior probability				Statistical power	*P*
									0.25	0.1	0.01	0.001		
Total	52	329035/730940	1.09 (1.06–1.12)	<.001	86.3	<.001	.592	100.00	1.48E‐09	4.44E‐09	4.89E‐08	4.93E‐07	1.000	4.94E‐10
Ethnicity														
European	24	288069/672710	1.08 (1.04–1.11)	<.001	88.3	<.001	.866	55.02	1.10E‐07	3.31E‐07	3.65E‐06	3.68E‐05	1.000	3.68E‐08
Asian	16	14029/15341	1.24 (1.13–1.37)	<.001	74.8	<.001	.018	17.86	7.05E‐05	2.11E‐04	0.002	0.023	1.000	2.35E‐05
Multiple	6	15177/26938	1.06 (0.94–1.18)	.351	89.8	<.001	.77	14.49	0.463	0.721	0.966	0.997	1.000	.287
African	2	7769/8643	0.99 (0.76–1.30)	.955	93.2	<.001	/	4.800	0.739	0.895	0.989	0.999	0.998	.942
African‐American	3	3317/5623	0.98 (0.75–1.28)	.889	93.9	<.001	.394	6.26	0.726	0.888	0.989	0.999	0.998	.882
Caucasian	1	674/1685	1.08 (0.92–1.26)	.337	0.0	/	/	1.57	0.496	0.747	0.970	0.997	1.000	.328
Cancer type														
Breast cancer	12	200498/227826	1.07 (1.03–1.11)	<.001	89.5	<.001	.941	32.08	0.001	0.003	0.029	0.232	1.000	3.02E‐04
Ovarian cancer	7	64225/137258	1.14 (1.10–1.19)	<.001	70.8	.002	.140	18.70	6.58E‐09	1.97E‐08	2.17E‐07	2.19E‐06	1.000	2.19E‐09
Lung cancer	5	7614/7844	1.19 (1.03–1.36)	.015	66.0	.019	.022	6.68	0.031	0.088	0.514	0.914	1.000	.011
Thyroid cancer	2	3453/288002	1.23 (1.11–1.38)	<.001	26.8	.243	/	3.70	0.001	0.004	0.040	0.296	1.000	4.22E‐04
RCC	2	536/879	1.29 (1.07–1.55)	.007	0.0	.432	/	1.65	0.020	0.059	0.407	0.874	0.946	.007
Prostate cancer	2	26413/28992	0.96 (0.67–1.37)	.832	97.9	<.001	/	5.42	0.716	0.883	0.988	0.999	0.978	.822
Pancreatic cancer	2	5752/8040	0.93 (0.87–0.99)	.031	0.0	.524	/	4.72	0.064	0.171	0.694	0.958	1.000	.023
Leukemia	2	3453/9023	1.12 (0.92–1.37)	.275	72.9	.055	/	3.88	0.448	0.709	0.964	0.996	0.998	.270
Colorectal cancer	2	2556/3215	1.10 (0.94–1.29)	.225	29.5	.234	/	3.06	0.420	0.684	0.960	0.996	1.000	.241
Glioma	2	2574/1828	1.18 (0.84–1.66)	.340	88.7	.003	/	3.05	0.528	0.771	0.974	0.997	0.916	.342
Other	14	11961/18033	1.06 (0.94–1.20)	.338	85.6	<.001	.122	17.06	0.517	0.763	0.973	0.997	1.000	.357
Cancer classification														
Gynecological cancer	19	264395/364026	1.11 (1.09–1.14)	<.001	82.0	.002	.050	50.15	5.13E‐14	1.54E‐13	1.69E‐12	1.71E‐11	1.000	1.71E‐14
Gastrointestinal cancer	8	10320/13468	1.21 (1.05–1.41)	.010	87.2	.035	.021	11.58	0.042	0.116	0.592	0.936	0.997	.015
Hematological tumor	7	7282/17241	0.97 (0.85–1.11)	.663	82.9	.023	.814	10.61	0.664	0.856	0.985	0.998	1.000	.658
Urinary tumor	5	30481/34991	1.04 (0.87–1.25)	.660	95.3	.036	.548	9.81	0.670	0.859	0.985	0.999	1.000	.676
Head and neck cancer	3	4308/289038	1.21 (1.14–1.29)	<.001	0.0	<.001	.667	4.95	1.61E‐08	4.82E‐08	5.30E‐07	5.35E‐06	1.000	5.35E‐09
Other	10	12249/12176	1.07 (0.93–1.22)	.340	82.6	.035	.843	12.90	0.484	0.737	0.969	0.997	1.000	.312
Source of control														
Multiple	27	304912/699583	1.06 (1.03–1.09)	<.001	90.8	<.001	.496	69.18	1.28E‐04	3.84E‐04	0.004	0.041	1.000	4.27E‐05
Population based	14	16857/25439	1.11 (1.04–1.18)	.003	61.9	<.001	.604	19.95	0.002	0.007	0.075	0.451	1.000	.001
Hospital based	11	5675/5918	1.24 (1.08–1.43)	.002	75.1	.001	.579	10.87	0.009	0.027	0.236	0.757	0.996	.003
Sample size														
Large	34	320240/720885	1.07 (1.04–1.10)	<.001	89.6	<.001	.551	83.67	4.86E‐06	1.46E‐05	1.60E‐04	0.002	1.000	1.62E‐06
Small	18	7204/10055	1.21 (1.11–1.33)	<.001	60.3	.001	.378	16.33	2.33E‐04	0.001	0.008	0.072	1.000	7.78E‐05
Method														
Illumina	25	244935/641683	1.07 (1.04–1.11)	<.001	90.9	<.001	.687	65.54	0.001	0.003	0.029	0.232	1.000	3.02E‐04
MassArray	12	6853/8458	1.24 (1.10–1.40)	.001	75.2	<.001	.21	12.64	0.002	0.005	0.048	0.339	0.999	.001
TaqMan	10	69956/73124	1.02 (0.93–1.12)	.639	77.6	<.001	.618	14.41	0.670	0.859	0.985	0.999	1.000	.678
Other	5	5700/7675	1.15 (1.08–1.22)	<.001	3.7	.386	.667	7.420	1.07E‐05	3.20E‐05	3.52E‐04	0.004	1.000	3.55E‐06

Note

*p* < .05 indicates statistical significance.

Abbreviations: 95% CI, 95% confidence interval; OR, odds ratio.

Stratification analysis identified increased cancer risk in subgroups of ethnicity in European (OR = 1.08, 95% CI: 1.04–1.11, *p*‐heterogeneity < .001, *I* ^2^ = 88.3%), Asian (OR = 1.24, 95% CI: 1.13–1.37, *p*‐heterogeneity = <.001, *I* ^2^ = 88.3%), multiple (OR = 1.06, 95% CI: 0.94–1.18, *p*‐heterogeneity = .351, *I* ^2^ = 89.8%), African (OR = 0.99, 95% CI: 0.76–1.30, *p*‐heterogeneity = .955, *I* ^2^ = 93.2%), African‐American (OR = 0.98, 95% CI: 0.75–1.28, *p*‐heterogeneity = .889, *I* ^2^ = 93.9%) and (OR = 1.08, 95% CI: 0.92–1.26, p‐heterogeneity = .337, *I* ^2^ = 0.0%) (Table 2). Subgroup analysis based on cancer type indicated that the *TERT* rs10069690 polymorphism was associated with an increased risk of breast cancer (OR = 1.07, 95% CI: 1.03–1.11, *p*‐heterogeneity < .001, *I* ^2^ = 89.5%), ovarian cancer (OR = 1.14, 95% CI: 1.10–1.19, *p*‐heterogeneity = .002, *I* ^2^ = 70.8%), lung cancer (OR = 1.19, 95% CI: 1.03–1.36, p‐heterogeneity = .019, *I* ^2^ = 66%), thyroid cancer (OR = 1.23, 95% CI: 1.11–1.38, *p*‐heterogeneity = .243, *I* ^2^ = 26.8%), and RCC (OR = 1.29, 95% CI: 1.07–1.55, *p*‐heterogeneity < .001, *I* ^2^ = 0.0%). No significant increase in risk was found in prostate cancer, leukemia, colorectal cancer, glioma and other cancers. However, a significantly decreased association was observed in pancreatic cancer (OR = 0.93, 95% CI: 0.87–0.99, *p*‐heterogeneity = .524, *I* ^2^ = 0.0%), as shown in Table 2.

Subgroup analysis based on cancer classification indicated that the *TERT* rs10069690 polymorphism was associated with an increased risk of gynecological cancer (OR = 1.11, 95% CI: 1.09–1.14, *p*‐heterogeneity < .001, *I* ^2^ = 82.0%), gastrointestinal cancer (OR = 1.21, 95% CI: 1.05–1.41, *p*‐heterogeneity = .035, *I* ^2^ = 87.2%) and head and neck cancer (OR = 1.21, 95% CI: 1.14–1.29, *p*‐heterogeneity < .001, *I* ^2^ = 0.0%). No significant increase in risk was found in hematological tumor, urinary tumor and other cancer (Table 2).A stratified analysis by source of controls indicated a significantly increased cancer risk in population based, hospital based, and multiple with ORs of 1.11 (95% CI: 1.04–1.18), 1.24 (95% CI: 1.08–1.43), and 1.06 (95% CI: 1.03–1.09), respectively. Moreover, a stratified analysis performed on the sample size revealed that the significant increased risk of cancer was also observed in large and small groups with ORs of 1.07 (95% CI: 1.04–1.10), 1.21 (95% CI: 1.11–1.33), respectively, as shown in Table 2. The stratified analysis based on Method of genotype indicated that the TERT rs10069690 polymorphism was associated with an increased risk of cancer in the Illumina (OR = 1.07, 95% CI: 1.04–1.11, *p*‐heterogeneity < .001, I^2^ = 90.9%) and MassArray groups (OR = 1.24, 95% CI: 1.10–1.40, *p*‐heterogeneity < .001, *I* ^2^ = 75.2%) (Table 2).

### 3.3 FPRP and statistical power

The FPRP values for significant findings at different prior probability levels are shown in Table 2. For a prior probability of 0.1, assuming that the statistical power was 1.00, the FPRP values were 4.44E‐09 for an association of rs10069690 allele with an increased risk of cancer. Positive associations with the rs10069690 observed in the subgroups of ethnicity (European and Asian), cancer type (breast cancer, ovarian cancer, lung cancer, thyroid cancer, RCC, and pancreatic cancer), cancer classification (gynecological cancer, gastrointestinal cancer, and head and neck cancer), source of control (PB and HB), sample size (large and small), and genotype method (Illumina and MassArray) were significant (Table 2).

### 3.4 Sensitivity analyses and publication bias

**Figure 3:**
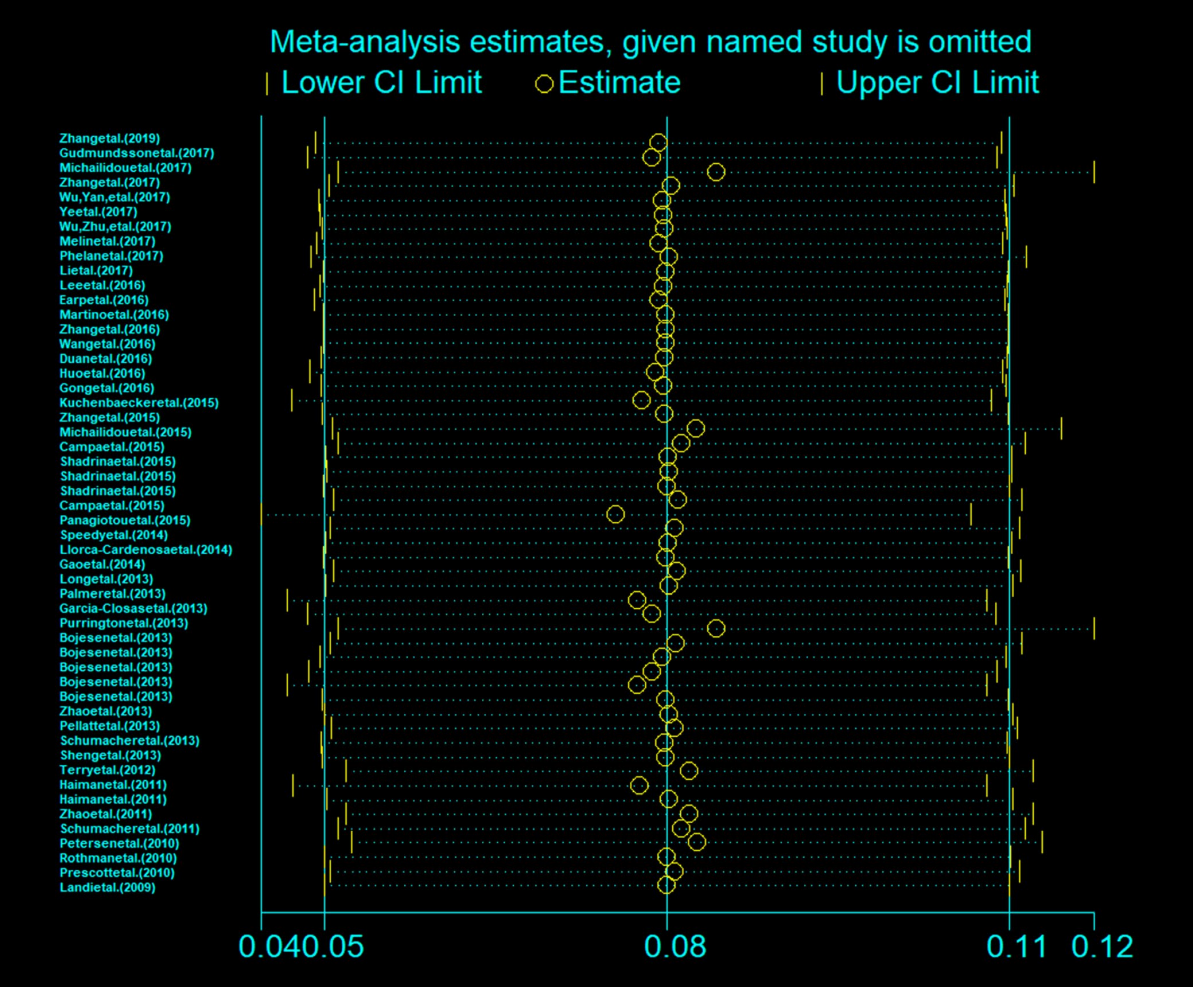
Sensitivity analyses of the overall ORs. The results were calculated by omitting each eligible study. Meta‐analysis random effects estimates were used

**Figure 4:**
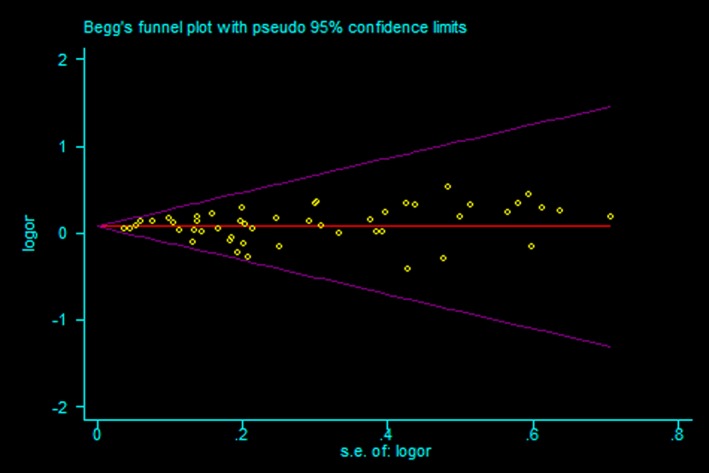
Begg's funnel plot for publication bias

Sensitivity analyses were performed to conclude whether modification of the inclusion criteria of the meta‐analysis affected the final results. The results showed that the significance of the OR was not affected by any single study (Figure 3). We used Begg's funnel plot and Egger's test to assess publication bias of the literatures. As shown in Figure 4, the shapes of the funnel plots seemed symmetrical and did not indicate any evidence of publication bias (*p* = .653). Egger's test results also did not show any evidence of publication bias (*p* = .592), indicating our results to be statistically robust.

## 4 DISCUSSION

A single nucleotide polymorphism (SNP) rs10069690 located in intron 4 of *TERT*, has been hypothesized to be associated with the risk of cancers development by many researchers, however, the results are conflicting and heterogeneous. Here, we performed a meta‐analysis included 45 case–control studies, including 329,035 cancer cases and 730,940 controls to explore the association between the *TERT* rs10069690 polymorphism and cancer risk. The result demonstrated that the *TERT* rs10069690 polymorphism was found to be associated with a significantly increased cancer risk overall. The association mainly existed in the European and Asian population, especially for breast cancer, ovarian cancer, lung cancer, thyroid cancer and RCC; but a significantly decreased association was observed in pancreatic cancer. In the subgroup analyses by cancer type, no significant association was found in prostate cancer, leukemia, colorectal cancer and glioma. The significant association between rs10069690 and cancer risk was also found in the stratification by cancer classification, source of controls, sample size, and genotype method.

*TERT* is mapped to chromosome 5p15.33 and consists of 16 exons and 15 introns spanning about 35 kb (Wick, Zubov, & Hagen, 1999). It encodes the catalytic protein subunit of telomerase and adds nucleotide repeats to chromosome ends in cooperation with a telomere RNA component (Cheung & Deng, 2008). A high level of TERT expression is involved in many tumors and it possibly contributes to unlimited cell division and carcinogenesis. The expression of the functional TERT protein is a prerequisite for acquisition of telomerase activity (Artandi & DePinho, 2000). Activation of telomerase has been implicated in human cell immortalization and cancer cell pathogenesis and telomerase expression is a key factor in cancer cell biology, enabling malignant cells to proliferate indefinitely (Greider, 1998). The biology of *TERT* makes it a compelling candidate gene for factors that influence cancer risk and *TERT* has been recognized as one of the most common tumor markers. A growing number of epidemiological studies have provided evidence that *TERT* polymorphisms contribute to cancer development (Jin et al., 2013; Li et al., 2012; Rafnar et al., 2009).

It has been reported that rs10069690 was associated with an increased risk of breast cancer (Bojesen et al., 2013; Haiman et al., 2011; Huo et al., 2016; Michailidou et al., 2015, 2017), ovarian cancer (Bojesen et al., 2013; Earp et al., 2016; Kuchenbaecker et al., 2015; Lee et al., 2016; Phelan et al., 2017), thyroid cancer (Gudmundsson et al., 2017), prostate cancer (Panagiotou et al., 2015), and glioma (Kinnersley et al., 2015; Melin et al., 2017; Ostrom et al., 2018; Rajaraman et al., 2012), through GWASs, but other studies have shown that the T allele was associated with a remarkably decreased risk of prostate cancer (Schumacher et al., 2011; Thomas et al., 2008), bladder cancer (Rothman et al., 2010), and testicular germ cell tumor (Schumacher et al., 2013). Additionally, a recent study composed of 386 patients and 495 controls suggested that the rs10069690 T allele was associated with increased risk of lung cancer (Ye et al., 2017), while other studies did not find any significant association between rs10069690 and risk of lung cancer (Gao et al., 2014; Landi et al., 2011; Wang et al., 2016). Other studies reported that the rs10069690 T allele was also not associated with risk of nasopharyngeal carcinoma (Zhang et al., 2016), melanoma (Llorca‐Cardenosa et al., 2014), colorectal cancer (Li et al., 2017), non‐Hodgkin's lymphoma (Prescott et al., 2010), and endometrial cancer (Burghaus et al., 2017; Prescott et al., 2010). As above, the results remain controversial and ambiguous.

The heterogeneity among studies in this meta‐analysis was significantly reduced in stratified analyses by the cancer type subgroups. These results suggested that the the role of polymorphism is potentially influenced by the tumor origins, and that stratified analysis is reasonable. Therefore, we can infer that rs10069690 had cancer‐specific contributions and may play different roles in the etiology of different tumor sites. More recently, a meta‐analysis study showed that rs10069690 polymorphism was associated with an increased breast cancer risk (Li, Dong, Feng, Zhang, & Cao, 2016). An agnostic subset‐based meta‐analysis (association analysis based on subsets) across six distinct cancers in 34,248 cases and 45,036 controls identified that rs10069690 T allele was positively associated with glioma, while being negatively associated with testicular, prostate, bladder and pancreatic cancer (Wang et al., 2014). The association between *TERT* rs10069690 polymorphism and longer telomere length has been recently reported (Pellatt, Wolff, Lundgreen, Cawthon, & Slattery, 2012). However, the exact biological function of rs10069690 has not been clarified until now. *TERT* rs10069690 polymorphism may contribute directly to disease predisposition by modifying the function of *TERT*, or it is in linkage disequilibrium (LD) with other disease‐causing mutations.

There are some limitations that should be addressed in interpreting the results of this meta‐analysis. First, due to insufficient genotype frequencies, we were unable to calculate the pooled ORs in other genetic models except allele model. Second, the origins of heterogeneity may include many factors, such as the ethnicity, cancer type, source of control, genotyping method and sample size. Finally, gene–gene and gene–environment interactions may have influenced our results, as cancer is mainly caused by genetic and environmental factors. In addition, the lack of detailed information, such as age and sex of the subjects, in some studies limited a more accurate OR would be corrected for age, sex and other factors that are associated with cancer risk.

## 5 CONCLUSIONS

The results of this meta‐analysis have shown that the *TERT* rs10069690 polymorphism is associated with an increased cancer risk overall. These results suggested that the *TERT* rs10069690 polymorphism may be a potential biomarker of cancer susceptibility. Overall, these results would help in understanding the role of this variant rs10069690 in cancer development and can aid in identifying new molecular targets focusing on cancer. However, the effect on cancer risk may be modified by ethnicity, cancer type, source of controls, sample size and genotype method. Considering the limitations of the present meta‐analysis, future studies with standardized unbiased methods, larger sample studies and well‐matched controls are required to validate the current findings and functional studies are warranted to reveal the role of the polymorphism rs10069690 in carcinogenesis.

## CONFLICT OF INTEREST

The authors declare that they have no conflict of interest.
